# Inter-laboratory comparison of 2 ELISA kits used for foot-and-mouth disease virus nonstructural protein serology

**DOI:** 10.1177/1040638720962070

**Published:** 2020-10-07

**Authors:** Clare F. J. Browning, Antonello Di Nardo, Lissie Henry, Tim Pollard, Lynne Hendry, Aurore Romey, Anthony Relmy, Phaedra Eble, Emiliana Brocchi, Santina Grazioli, Donald P. King, Anna B. Ludi

**Affiliations:** The Pirbright Institute, Pirbright, Surrey, United Kingdom; The Pirbright Institute, Pirbright, Surrey, United Kingdom; The Pirbright Institute, Pirbright, Surrey, United Kingdom; Animal and Plant Health Agency, New Haw, United Kingdom; Animal and Plant Health Agency, New Haw, United Kingdom; ANSES, Laboratoire de Santé Animale de Maisons-Alfort, INRA, École Nationale Vétérinaire d’Alfort, ANSES, Université Paris-Est, Maisons-Alfort, France; ANSES, Laboratoire de Santé Animale de Maisons-Alfort, INRA, École Nationale Vétérinaire d’Alfort, ANSES, Université Paris-Est, Maisons-Alfort, France; Wageningen Bioveterinary Research (WBVR), Lelystad, The Netherlands; Istituto Zooprofilattico Sperimentale della Lombardia e dell’Emilia-Romagna, Brescia, Italy; Istituto Zooprofilattico Sperimentale della Lombardia e dell’Emilia-Romagna, Brescia, Italy; The Pirbright Institute, Pirbright, Surrey, United Kingdom; The Pirbright Institute, Pirbright, Surrey, United Kingdom

**Keywords:** ELISA, foot-and-mouth disease virus, inter-laboratory study, serology

## Abstract

Serologic assays used to detect antibodies to nonstructural proteins (NSPs) of foot-and-mouth disease virus (FMDV) are used for disease surveillance in endemic countries, and are essential to providing evidence for freedom of the disease with or without vaccination and to recover the free status of a country after an outbreak. In a 5-site inter-laboratory study, we compared the performance of 2 commercial NSP ELISA kits (ID Screen FMD NSP ELISA single day [short] and overnight protocols, ID.Vet; PrioCHECK FMDV NS antibody ELISA, Thermo Fisher Scientific). The overall concordance between the PrioCHECK and ID Screen test was 93.8% (95% CI: 92.0–95.2%) and 94.8% (95% CI: 93.1–96.1%) for the overnight and short ID Screen incubation protocols, respectively. Our results indicate that the assays (including the 2 different formats of the ID Screen test) can be used interchangeably in post-outbreak serosurveillance.

Foot-and-mouth disease (FMD), caused by foot-and-mouth disease virus (FMDV; *Picornaviridae*), is an economically devastating livestock disease affecting domesticated cloven-hoofed animal species such as cattle, sheep, and pigs.^4^ In endemic countries, the economic costs associated with FMD are estimated to be US$6.5–21 billion annually,^5^ with outbreaks in FMD-free countries and zones potentially causing economic losses of >$1.5 billion.^6^

Therefore, countries free from the disease have contingencies in place that can be deployed in the event of an outbreak. These contingency plans outline steps that will be taken to regain FMD-free trading status, following the guidelines provided by the World Organisation for Animal Health (OIE, https://www.oie.int/standard-setting/terrestrial-manual/access-online/). Prior to lifting restrictions, these OIE guidelines specify that serosurveillance should be undertaken to identify whether seropositive animals are present within herds. ELISA kits that detect antibodies to nonstructural proteins (NSPs) of FMDV are widely used for this purpose, and are also a useful tool to monitor the distribution of FMD in endemic countries.^8^ Comparisons of different ELISA kits have been carried out in the past;^1,2,7,9^ however, most of the kits used for these studies are no longer available, or have now been acquired by different companies, and newer commercial kits are also available on the international market.

To compare the performance and assess reproducibility and repeatability of 2 NSP ELISAs on the market at the time of publication (PrioCHECK FMDV NS antibody ELISA kit; Thermo Fisher Scientific; ID Screen FMD NSP competition, ID.Vet), 5 ISO/IEC 17025–accredited (https://www.iso.org/standard/66912.html) European laboratories were selected, with 2 operators from each laboratory receiving their own separate blind and randomized panel for testing. The panel consisted of 180 sera (90 positive and 90 negative) from bovine, ovine, and porcine species (60 sera for each species) that were representative of samples that might be collected from 1) naïve, 2) infected, or 3) vaccinated and infected animals. Sample status was defined based on the FMDV-infected vs. FMDV non-infected status of the animal and by initial results obtained using the PrioCHECK NSP ELISA and the ID Screen (overnight and short) ELISAs (Fig. 1). The negative samples (30 of each bovine, ovine, and porcine) were collected from naïve animals from an FMDV-free country. The positive sera of 30 bovine, 30 ovine, and 5 porcine animals infected at various times after vaccination and infection (with a minimum of 8 days post-infection [dpi]) were sourced from previous experimental studies conducted at The Pirbright Institute (Surrey, U.K.). A porcine NSP-positive serum (O Taiwan 97 Animal 6533, 52 dpi) was sourced from experimentally infected animals at the Wageningen Bioveterinary Research (WBVR; Lelystad, The Netherlands). This sample was pre-titrated to enable the generation of 25 positive porcine samples (4 diluted 1 in 4, 11 diluted 1 in 8, and 10 diluted 1 in 10).

**Figure 1.**
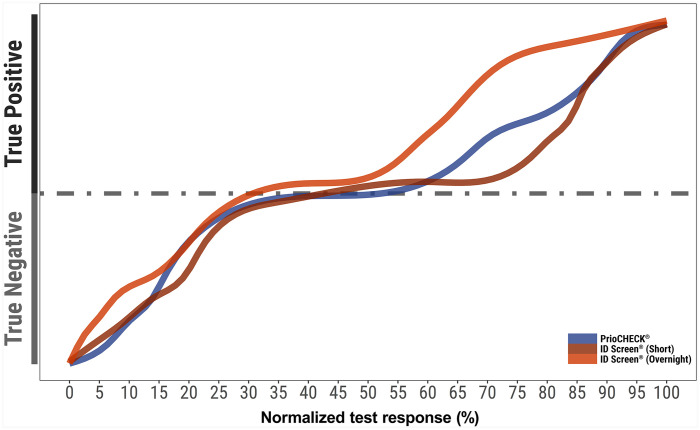
Status of samples used in the study of 2 FMDV NSP ELISA kits. Results were obtained from testing the panel of sera (*n* = 180) with the ELISAs used in the inter-laboratory exercise. The true-negative and true-positive status of the serum (y-axis) was defined by the history of the animal. The test response was calculated: PI(100 – [mean sample OD/mean OD of negative control] × 100) for the PrioCHECK results, and normalized sample-to-negative ratio for the ID Screen assays: (100 – [mean sample OD/mean OD of negative control] × 100).

Samples (bromoethyleneimine-inactivated sera) were shipped on dry ice to ensure that they were only thawed once upon testing. The PrioCHECK kits were shipped from the organizing laboratory (The Pirbright Institute) on cold packs alongside the sample panels; the ID Screen kits were shipped directly from the manufacturer. All laboratories received the same batch of kits for each of the ELISAs. Each operator followed the protocol provided by the manufacturer including overnight and short protocols for the ID Screen test (in which the sera were tested at a dilution of 1/10 and 1/2.6 for the overnight and short methods, respectively). The short protocol has a sample incubation step of 2 h at 37°C (± 3°C) compared to the overnight protocol of 16–20 h at room temperature (21 ± 5°C). The PrioCHECK kit only provides for an overnight sample incubation at room temperature (23 ± 3°C). All samples were tested in duplicate, and the kit control samples were included on every test plate. The PrioCHECK kit contained one each of a positive, weak-positive, and negative control; the ID Screen kits included a positive and a negative control only. The operators were given a testing schedule and plate-plan, and then asked to complete all of the testing over 3 consecutive days to reduce variability resulting from storage conditions of the serum samples.

Results were submitted from the participating laboratories and included individual well raw optical density (OD) data, as well as the interpretation of each sample (mean percentage inhibition [PI = 100 – serum/negative × 100%] for the PrioCHECK or competition percentage [serum/negative %] for ID Screen). However, given the similarity of test principles, the ID Screen assay results were normalized and reported as PI for a more direct comparison of results.

The intra-laboratory reproducibility (i.e., the likelihood of obtaining similar results from different operators within the same laboratory) and the inter-laboratory reproducibility (i.e., the likelihood of obtaining similar results from different laboratories and the interaction between operators within the same laboratory) was calculated for each test.^3,10^ The coefficient of variation (CV) measured the dispersion of estimated parameters across different laboratories, and the Cochran Q test considered differences between results generated by different laboratories. Differences between results generated by different operators were assessed using the McNemar test. ANOVA analysis following logit model fit evaluated the contribution of testing performed by different laboratories and operators in the variability of the results generated by each of the tests in a single analysis. The Cohen kappa statistic test compared the level of agreement between the PrioCHECK and each of the ID Screen tests (Fig. 2).^3^

**Figure 2.**
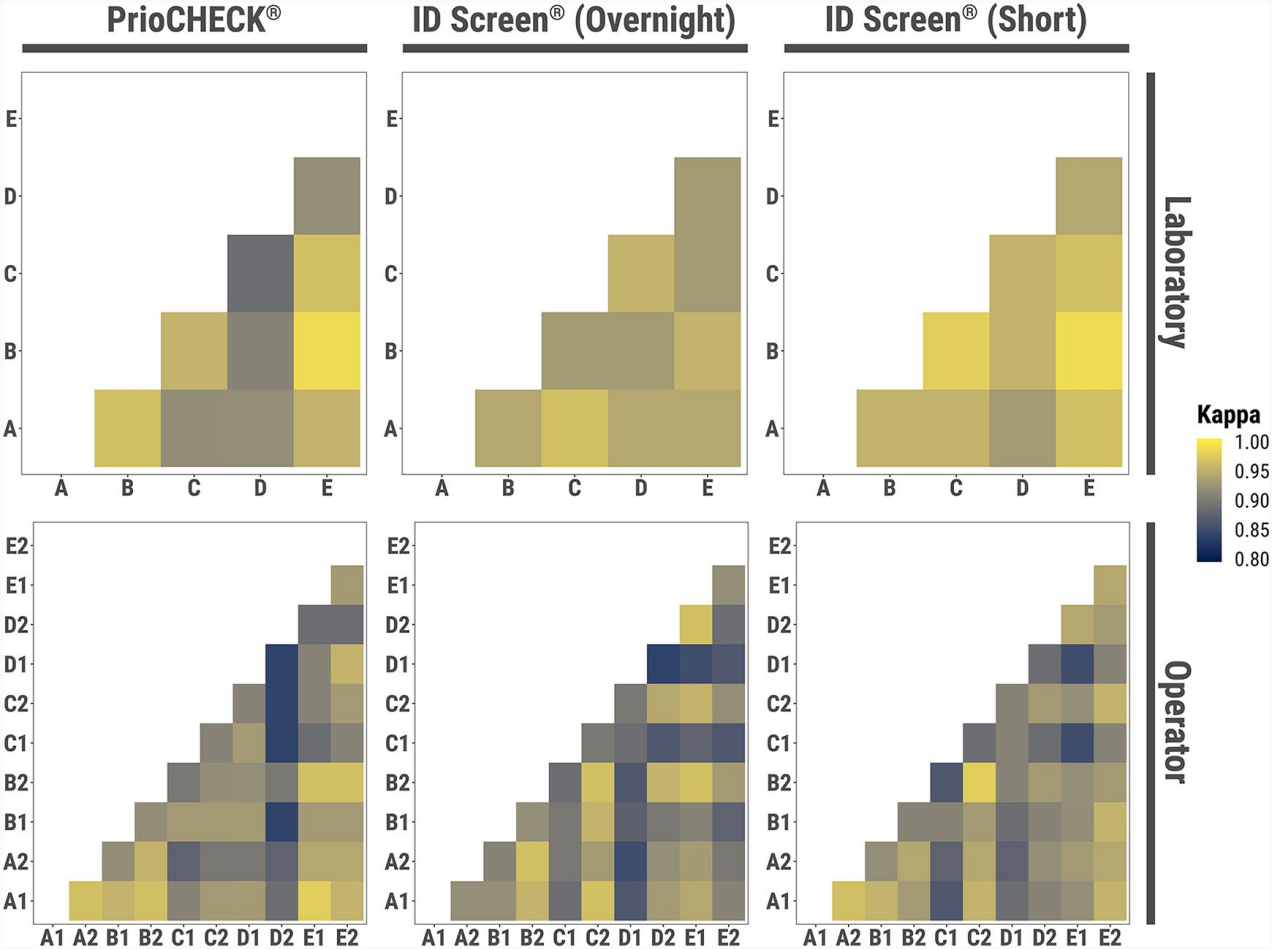
Comparative analysis of the operators and laboratories for each of the FMDV NSP ELISAs. The Cohen kappa statistic test was used to analyze the variation between laboratories and operators. For the top panels, the x- and y-axes represent the 5 laboratories. For the bottom panels, the x- and y-axes represent the 2 operators from each laboratory (denoted A–E). Colors denote concordance between operators or laboratories.

Variability of results between laboratories was minimal, and simple CV analyses generated measures of 0.6% (95% CI: 0.4–2.6%) for sensitivity and 1.4% (95% CI: 0.8–5.4%) for specificity. There was no reported statistical difference between the results generated by each of the laboratories using either the PrioCHECK (Q_348,4_ = −0.230, *p* > 0.05) or the ID Screen (overnight: Q_348,4_ = −0.192, *p* > 0.05; short: Q_348,4_ = −0.05, *p* > 0.05), which indicated a high level of testing reproducibility in different laboratory settings. Repeatability of results by different operators was found to vary only on repeated testing using the ID Screen overnight (χ^2^ = 5.818, *p* = 0.023). When assessing the reproducibility of test results, no statistical evidence was found for the contribution of the laboratory (χ^2^ = 1.19, *p* = 0.879), the operator (χ^2^ = 0.37, *p* = 0.542), or their interaction (χ^2^ = 1.03, *p* = 0.905) in the variability of results.

The overall concordance between the PrioCHECK and ID Screen test was 93.8% (95% CI: 92.0–95.2%) and 94.8% (95% CI: 93.1–96.1%) for the overnight and short incubation protocols, respectively (Fig. 3, Table 1). On initial analysis, 6 false-positive samples were identified (3 bovine and 3 ovine). These samples were from naïve animals that were collected from a FMDV-free country that were negative on initial screening (data contributing to Fig. 1); however, they were positive by all operators on all assays when assessed as part of the inter-laboratory exercise. Follow-up experiments suggest that storage conditions may have been a contributing factor toward the false-positive status of these samples, and heat inactivation to 56°C for 30 min could reassign their status to negative. These unexpected observations may have particular impact on serologic studies that use sera that are refrigerated for long time periods. The results from these 6 animals were not included in the statistical analysis.

**Figure 3.**
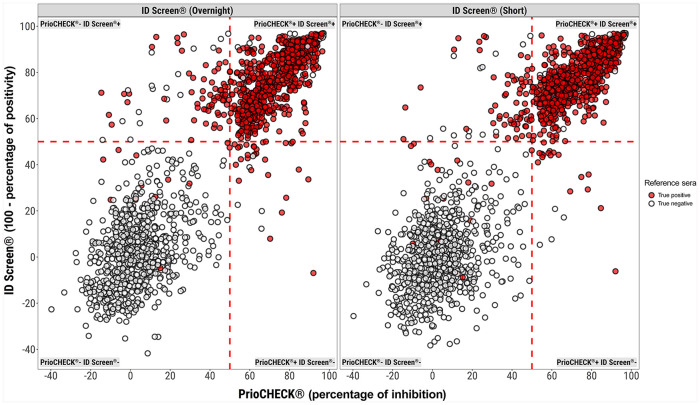
Concordance of the results of the 2 ID Screen FMDV NSP ELISA kits with the PrioCHECK ELISA. The mean is plotted of duplicate determinations by each operator in the participating laboratories. The true positives (red) and the true negatives (open circles) were determined by the animal status. The left panel compares the ID Screen overnight (y-axis) and the PrioCHECK (x-axis). The right panel compares results for the ID Screen short against PrioCHECK. The red dashed lines define the cutoff for each assay.

**Table 1.** Agreement between FMDV NSP serologic tests.

Test	OPA	PPA	NPA	κ	Lab CV for κ	Opr CV for κ
PrioCHECK vs. ID Screen (overnight)	93.8 (92.0–95.2)	88.5 (85.3–91.0)	88.1 (85.0–90.7)	0.88 (0.84–0.91)	4.6 (2.7–12.6)	4.1 (2.8–8.0)
PrioCHECK vs. ID Screen (short)	94.8 (93.1–96.1)	90.4 (87.4–92.7)	89.9 (86.7–92.3)	0.90 (0.87–0.92)	3.0 (1.8–8.3)	4.1 (2.8–8.0)
ID Screen (overnight) vs. ID Screen (short)	97.8 (96.6–98.6)	95.7 (93.4–97.2)	95.7 (93.5–97.2)	0.96 (0.94–0.98)	1.6 (1.0–5.9)	3.0 (2.0–5.7)

κ = Cohen kappa statistic test; Lab CV for κ = laboratory coefficient of variation; NPA = negative percentage of agreement; OPA = observed percentage of agreement; Opr CV for κ = operator coefficient of variation; PPA = positive percentage of agreement. Numbers in parentheses are 95% CIs.

The observed percentage of agreement (OPA) between the PrioCHECK and the ID Screen tests (overnight and short) was relatively high, with slightly better performance observed with the short incubation protocol (Table 1). The assessment of test comparison performed using the kappa statistic (κ) provides evidence of almost perfect agreement (>0.8) for both of the ID Screen tests with the PrioCHECK. Kappa estimates were consistent across different laboratories, with 4.6% (95% CI: 2.7–12.6%) and 3.0% (95% CI: 1.8–8.4%) of differences in the results obtained with the ID Screen (overnight) and (short) tests, respectively (Fig. 3, Table 1). Similar variability was also observed in estimates obtained by different operators, with 4.1% (95% CI: 2.8–8.0%) difference in the results. The agreement of ID Screen tests (overnight vs. short) were found to be higher than the single test comparison with PrioCHECK, with a κ estimate of 0.96 (95% CI: 0.94–0.98). Inter-laboratory and inter-operator variabilities was also lower when comparing the ID Screen kits, with 1.6% and 3.0% difference in the results, respectively.

Our objective was to assess the concordance between the NSP tests, rather than define the specificity and sensitivity of the individual assays. We found the PrioCHECK and ID Screen NSP kits to be reproducible between operators and laboratories. Our findings indicate that these tests can be used interchangeably, which is particularly useful, given that during the 2001 U.K. outbreak, ~3 million blood samples were tested for FMDV antibodies by ELISA.^8^ This type of operation requires the scaling-up of resources including the procurement of a large number of kits. If the 2001 outbreak samples had been tested by the current ELISAs, ~8,000 kits would need to have been purchased, placing a strain on companies supplying these kits. Therefore, having multiple commercial kits, such as the kits in our study, with equivalent performance within FMD reference laboratories, is ideal.
